# Repeated Galvanic Vestibular Stimulation Modified the Neuronal Potential in the Vestibular Nucleus

**DOI:** 10.1155/2020/5743972

**Published:** 2020-05-27

**Authors:** Gyutae Kim, Sangmin Lee, Kyu-Sung Kim

**Affiliations:** ^1^Research Institute for Aerospace Medicine, Inha University, Incheon, Republic of Korea; ^2^Department of Electronic Engineering, Inha University, Incheon, Republic of Korea; ^3^Department of Otolaryngology Head and Neck Surgery, Inha University Hospital, Incheon, Republic of Korea

## Abstract

Vestibular nucleus (VN) and cerebellar flocculus are known as the core candidates for the neuroplasticity of vestibular system. However, it has been still elusive how to induce the artificial neuroplasticity, especially caused by an electrical stimulation, and assess the neuronal information related with the plasticity. To understand the electrically induced neuroplasticity, the neuronal potentials in VN responding to the repeated electrical stimuli were examined. Galvanic vestibular stimulation (GVS) was applied to excite the neurons in VN, and their activities were measured by an extracellular neural recording technique. Thirty-eight neuronal responses (17 for the regular and 21 for irregular neurons) were recorded and examined the potentials before and after stimulation. Two-third of the population (63.2%, 24/38) modified the potentials under the GVS repetition before stimulation (*p* = 0.037), and more than half of the population (21/38, 55.3%) changed the potentials after stimulation (*p* = 0.209). On the other hand, the plasticity-related neuronal modulation was hardly observed in the temporal responses of the neurons. The modification of the active glutamate receptors was also investigated to see if the repeated stimulation changed the number of both types of glutamate receptors, and the results showed that AMPA and NMDA receptors decreased after the repeated stimuli by 28.32 and 16.09%, respectively, implying the modification in the neuronal amplitudes.

## 1. Introduction

Vestibular compensation is the functional recovery after partial or complete damages of the semicircular canals or the macular receptors in an inner ear [1–3]. Generally, no peripheral vestibular loss is physically reconstructed, and the damage causes various functional problems, such as vertigo, gait unsteadiness, and oscillopsia [4]. Thus, the functional recoveries are only possible through the neural plasticity by strengthening the neural network or increasing the neuronal connectivity. The neural basis for compensation has been thoroughly investigated mostly by vestibulo-ocular reflex (VOR) in different species [5–9]. Based on the early examinations for VOR, several candidates of central regions were proposed for the vestibular neural plasticity, such as in the cerebellar flocculus [10] and the brain stem [11], and accumulated evidences confirmed them as the possible central areas for the vestibular neuroplasticity [12]. Unlike previous lesion studies, however, it has been little reported how the neuroplasticity was generated and how to assess its related neural activities in the central vestibular system. This question is critical to understand the artificially induced neuroplasticity for the treatments of neurological disorders, such as Parkinson's disease [13, 14]. Furthermore, the study of the induced neuroplasticity is closely related with the mechanism of current medical treatments using an electrical stimulation, such as deep brain stimulation (DBS) [15] or galvanic vestibular stimulation (GVS) [13, 16].

Neuroplasticity modifies the strength of synaptic weights [17]. Thus, the modifications in neuronal potentials highly suggest the concurrent neural reorganization by reweighting the connectivity among individual neurons. Depending on the lasting activity, the modified potential is generally classified as short- and long-term potentiation or depression, and it has been known that their mechanisms are different with no interruption to the other's process [18]. On the other hand, the neural activity of sensory systems often requires fast projection on different nervous areas to generate their functional responses on time [19]. Considering that the vestibular system processes incoming kinetic stimulations in a short period of time, its short responding characteristics are fundamental for the vestibular neuroplasticity. Moreover, the neuroplasticity by electrical stimulation was more intuitively recognized than that by motor trainings in previous studies [20–22]. A previous study had observed the increased number of glutamate receptors as an electrical stimulus was repeated [23]. Due to its critical role for plasticity, the change in the number of glutamate receptors suggested the indication that the neural plasticity was induced by the repeated electrical stimulation within a short period of time [24].

Here, the neural plasticity induced by electrical stimulation was investigated. To identify the artificially induced plasticity, the vestibular system was used as the targeted area, specifically the vestibular nucleus (VN), and the guinea pigs with well vestibular function were adopted in the overall experiments. The electrical stimulation (GVS) was applied in the bipolar and bilateral configuration, and the detail approach was maintained as done in our previous studies [25–27]. Even though the short distance between the stimulated area and VN served as the pretext in excluding VN for the vestibular plasticity, the potential modification in VN has been implied in many previous studies [28–31]. Moreover, the revealed relations between plasticity and stimulation demonstrated the high efficiency to produce the plasticity by different types of electrical stimulation [28–31]. Because of the increasing importance in neurorehabilitation, an electrical stimulation on the brain has been a core approach to trigger the plasticity for the rehabilitation. However, the neural effects for the plasticity by electrical stimulations have been generally shown in the behavioral level, missing the concurrent neural responses in vivo. Thus, our current study is expected to provide the possible neural activity related with the plasticity by the repeated electrical stimulations.

## 2. Materials and Methods

Guinea pigs were used for this study, and all procedures and principles of laboratory animal care were approved by the Animal Ethics Committee at Inha University. A total of 29 animals (7-10 weeks old, male) were used for neural recording, and all animals were supplied from Orient Bio (Korea). Animals were cared in Inha University Animal Care Center with consistent environmental parameters, such as temperature, humidity, illumination, and noise.

### 2.1. Animal Preparation and Experimental Setup

An animal was anesthetized by the solution of ketamine and Rompun (IM injection, 1.3 ml/kg). Following anesthesia, the animal's head and body were fixed in a motorized stereotaxic system (NEUROSTAR, Deutschland), and its superior part of the skull was exposed. Using the cross point by the line of two external auditory canals and the sagittal suture line, a center position was designated on the skull, and a 2.0 mm diameter hole was made located in 2-3 mm posterior and 1.5-2 mm lateral positions away from the center. The hole was used for the entrance of the recording electrode, and its position was always right based on the sagittal suture line. Once the animal was ready for the neuronal recordings, the stereotaxic apparatus was loaded on a rotatory table, and the neuronal response to the stimulation of rotation was made possible to test.

### 2.2. Neural Recording

Overall neural recording technique followed our previous methods [25–27]. In short, the extracellular neural recordings in VN were conducted using epoxy-coated tungsten electrodes (A-M Systems, WA). The impedance of the electrodes was ranged between 5 and 12 M*Ω*, and its length was 127 mm. While advancing the electrode through the hole, the recording area was identified based on the neuronal activities. In the brain of a guinea pig, VN was located below the cerebellum, which provided the unique firing characteristic of complex spikes. Depending on the neuronal activities, the position of the electrode was estimated. In addition, three-dimensional (3D) coordinates of the position of the electrode were continuously measured, and they were evaluated off-line. When the neuronal response to the head rotation was identified, the movement of the electrode was ceased, and the neuron was tested by horizontal rotation and electrical stimulation. In this study, all neurons responded to both types of stimulation, indicating that their responding origins were from the full or partial activity by the horizontal semicircular canal. Confirming the neurons in VN, two main approaches were adopted during on/off-line. During on-line session, the kinetic and the electrical stimulations were applied to identify the concurrently occurring neuronal responses, and the referenced [32] and measured recording locations were compared during off-line session. The neuronal responses to the horizontal rotation and GVS were also analyzed in off-line, and if the recorded neurons were those in VN was finally confirmed.

### 2.3. Galvanic Vestibular Stimulation (GVS)

To stimulate the neurons in VN, a direct current (DC) was applied. The iron wire with the excitatory polarity was attached to one side and that with the inhibitory polarity to the other side of the ears [25–27]. The applied electrical stimulation was mainly characterized by three parameters: the amplitude of current, its lasting duration, and the interval between two successive stimuli. To generate a stimulus with appropriate values of the parameters, the possible values for each parameter were evaluated. First, the amplitude was examined based on the instantaneous firing rates (IFR) by applying different amplitudes (range: 100-500 *μ*A, resolution: 50 *μ*A). For each amplitude of current, the aligned responding IFR were calculated. Based on the amplitudes and the IFR, the relation between the electrical strength and the neuronal response was obtained. Second, the duration of stimulation was determined, avoiding any chance of synaptic fatigue, which was a temporary inability of a neuron to fire. When a neuron was electrically stimulated for an excessive duration, the neuron would not fire at a normal rate. To find the excessive time and an appropriate period of stimulation, the neuronal responses was investigated during different stimulating periods (1-20 sec). Third, the interval between two successive electrical stimulations were determined to allow a stimulated neuron to recover its IFR before the next stimulation. For this purpose, the recovery of the neuronal IFR after stimulation was examined. During the repeated stimulations with the same size of amplitude, two averaged IFRs before each stimulation were compared to assess if the neuronal responses were affected by the previous stimulation. The overall examination determined GVS with a specific strength (100 *μ*A), a constant interval (3 sec), and the number of repetition (5 times). GVS polarity was adopted when it increased the neuronal IFR. Two successive GVS were separated by the interval (60 sec) which rarely affected the neuronal responses.

### 2.4. Data Analysis

All data analyses were performed off-line using a custom-made software written in MATLAB language (MathWorks, Cambridge, MA). The characteristics of the neurons were calculated or recorded: the discharge regularity, the directional preference (DP), the polarity of GVS for the neuronal excitation (GP), the neuronal sensitivity to GVS (SE), and the physiological response (PR). The discharge regularity was calculated using the relation between the mean interspike interval (ISI) and the coefficient of variation (CV). The mean ISI was obtained by averaging time intervals in two successive spikes. As previous studies suggested, the CV was normalized (CV^∗^) to avoid the variance in time [20]. Using the neuronal activity during an initial 1-second resting period, the mean (*μ*) and standard deviation (*σ*) of interspike interval (ISI) were obtained, and the coefficient of variation (CV) was computed as follows:

(1) $$\begin{array}{c} \text{CV}=\frac{\sigma }{\mu }. \end{array}$$

The CV^∗^ was again calculated by a formula modified from a previous study [33].

(2) $$\begin{array}{c} {\text{CV}}^{*}=10\times {\left[\frac{\text{CV}}{0.7116\log\left(\mu \right)-0.8248}\right]}^{1/\left(0.00002\mu^{3}-0.0024\mu^{2}+0.0731\mu +0.37\right)}. \end{array}$$

Then, the neurons were classified as regular (CV^∗^ < 0.15) and irregular (CV^∗^ > 0.15) neurons. The DP and the GP were tested during the recording session. A neuron was defined as the neuron with ipsi-DP if the neuronal IFR increased as the head moved to the right side during the horizontal head rotation (see Animal Preparation and Experimental Setup). In the opposite case, the neuron was classified as a neuron with contra-DP. The GP was determined by the neuronal excitation. In addition, the PR was evaluated by the IFR consistence during GVS. If the IFR was constant during the stimulation, the neuron was classified as a tonic neuron, and a neuron was phasic if its IFR gradually decreased or increased during GVS. In final, the SE was calculated using the initial averaged IFR in resting and stimulation, as follows:

(3) $$\begin{array}{c} \text{SE}=\frac{\left(\text{mean IFR in GVS}-\text{mean IFR in resting}\right)}{\text{mean IFR in resting}}. \end{array}$$

Based on the calculated SE, the neurons were divided into high- and low-sensitive groups; if the SE of a neuron was more than 1, which meant the mean IFR during stimulation was two times more than its mean IFR during the resting period, the neuron was counted as a high-sensitive neuron. Otherwise, the neuron was considered as a low-sensitive neuron.

The stimulation and its neuronal response were aligned in time, and the IFR was presented to show the effects by GVS. For each neuron, the neuronal spikes for 1 second before and after GVS were separately collected, and the collected spikes were superimposed within the same time domain. Using the overlapped spikes, an averaged spike was obtained, and its peak-to-peak (P-P), the difference between the minimal and maximal values of the averaged spike, was calculated. The averaged ISI was also calculated based on the collected spikes in the duration (1 sec). Therefore, the neuronal potential was mainly investigated in the P-P of the averaged spike, and the modification in the ISI was used to identify the constant neuronal responses during the change in the P-P. Including only sorted spikes, any possible effects by noises or other concurrently responding neurons were minimized.

Depending on the changing rate in the P-P, the neuronal modification was classified into three responding categories: potentiation (rate of change > 1 *μ*V/sec), depression (rate of change < −1 *μ*V/sec), and no modulation (−1 *μ*V/sec < rate of change < 1 *μ*V/sec). Of the three groups, the neurons included in the categories of potentiation and depression were counted as those with neuronal modulation. The stimulation was applied at every 60 seconds up to five times, and the slope by a linear regression on the P-P indicated the changing rate in the P-P and the ISI for 300 seconds (see Results). For the analysis of the population, the values of P-P responding to each stimulation was normalized (Equation (4)), and their averages and standard deviations were calculated. The same method was used for the population of neuronal ISI (Equation (5)), and if the ISI was maintained during GVS was examined, which might affect the modification of the neuronal potentials.

(4) $$\begin{array}{c} \text{normalized }{\left(\text{P}-\text{P}\right)}_{i}=\frac{{\left(\text{P}-\text{P}\right)}_{i}}{\sqrt{\sum_{k=1}^{5}{\left|{\left(\text{P}-\text{P}\right)}_{k}\right|}^{2}}}, \end{array}$$

(5) $$\begin{array}{c} \text{normalized }{\left(\text{ISI}\right)}_{i}=\frac{{\left(\text{ISI}\right)}_{i}}{\sqrt{\sum_{k=1}^{5}{\left|{\left(\text{ISI}\right)}_{k}\right|}^{2}}}, \end{array}$$

where P-P and ISI were the peak-to-peak of the averaged neuronal spike and the averaged interspike interval between two successive spikes for 1 second, respectively. The subscript, *i*, denoted the *i*^th^ P-P or ISI, and the stimulation was repeated up to five times.

### 2.5. Immunohistochemistry

**Figure 1:**
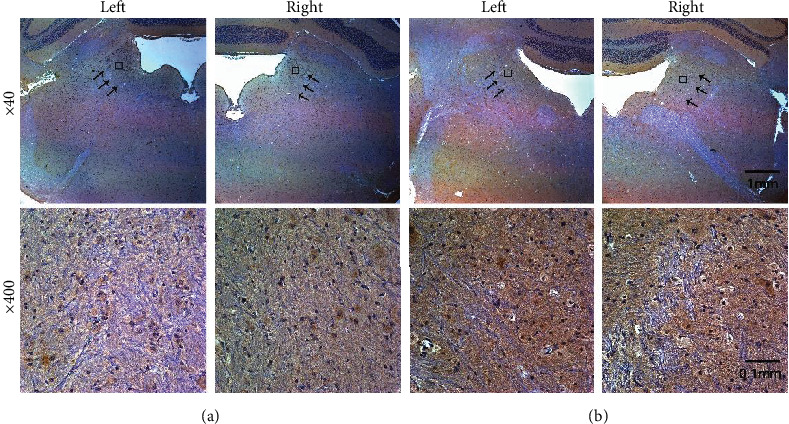
Histological results for *α-amino-3-hydroxy-5-methyl-4-isoxazolepropionic acid* (AMPA). The control (a) and the stimulated slices (b) are prepared for the immunohistochemistry. The arrowed areas at the upper part indicate the vestibular nucleus (VN) where the captured receptors are counted. The rectangle at the upper indicates the enlarged image at the bottom. Compared with the counted receptors before and after stimulations, the number of the receptors decreases. The picture at the bottom represents one of the arrowed areas. The upper figures are expanded 40 times, and the bottoms are 400 times of the originals.

**Figure 2:**
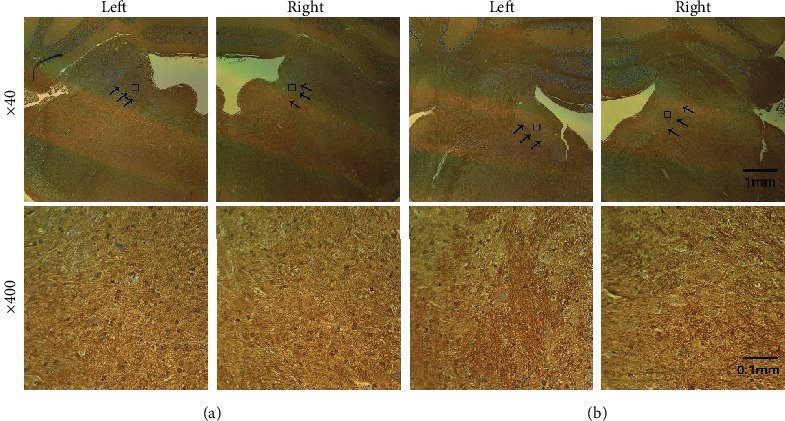
Histological results for *N-methyl-D-aspartate* (NMDA). The overall format is same as explained in Figure 1. The control (a) and the stimulated slices (b) are prepared for the immunohistochemistry. The black arrows at the upper implies the areas to count the number of the receptors. The rectangle at the upper indicates the enlarged image at the bottom. The picture at the bottom represents one of the arrowed areas. The upper pictures are expanded 40 times, and the bottoms are 400 times of the originals.

The number of the glutamate receptors, *α-amino-3-hydroxy-5-methyl-4-isoxazolepropionic acid* (AMPA) and *N-methyl-D-aspartate* (NMDA), before and after stimulation was assessed by immunohistochemistry, and its overall processes were as follows. First, an animal was sacrificed after the repeated electrical stimulations, and its removed brain was fixed within 10% formalin for less than 72 hours. The fixed brain was cut to separate VN, and its paraffin block was made. Then, the block embedded the tissue was sliced by 5 *μ*m thick and dried in 60°C. To detect AMPA and NMDA receptors in the sliced sections, two retrieval solutions of antigens were prepared; pH 9.0, Tris-EDTA 95°C 50 min, and pH 6.0, Citrate sol. 95°C 50 min, respectively. The sections were visualized with synthetic mounting medium, and the final images were captured by the Axioplan 2 imaging system (Carl Zeiss Meditec, Germany). The receptors were detected by their subunits, GluR1 (PA1-46151, Thermo Fisher Scientific, US) and GluN1 (PA3-102, Thermo Fisher Scientific, US), for AMPA and NMDA receptors, respectively. The number-counting for each kind of receptors was performed with the expanded figures (Figures 1 and 2, bottoms) by selecting three areas of VN. The captured receptors were independently counted, and their average and standard deviation (STD) were calculated to assess the change in the number. For the control, an animal undergone the same procedure except the stimulation.

### 2.6. Statistical Test

Using the number of neurons classified in the characteristics, the statistical significance was tested (binomial cumulative distribution, BCD). Its main purpose was to examine if the number of neurons in each group of characteristics had any significance related with the modification of the P-P. Based on the classified neurons as the affected (potentiation and depression) and unaffected P-P (no modulation), therefore, the test was expected to provide a significance for the neuronal characteristic in a 50% chance. If the test resulted in a significance (*p* − value < 0.05), the modification of the P-P could be influenced by a specific neuronal characteristic. In addition, the same method was applied on the groups of the neuronal characteristics, such as DP, SE, GP, and PR, in both regular and irregular neurons to test the relation between the discharge regularity and the neuronal characteristics. This analysis was also used to test the significance of the number of the modified potentials in the population.

To examine the effects on the potential modulation by the neuronal ISI, their relations were tested. A linear regression was applied on the relation plot between the ISI and the P-P, and the R-squared was calculated. The regression was applied on both neuronal responses before and after stimulations, and the significance was determined when both R-squared values satisfied a general interpreting rule [34]. Based on the general value of R-squared, 70%, the linear relation between the ISI and the P-P was examined, and the effects of the ISI on the P-P were concluded.

## 3. Results

**Figure 3:**
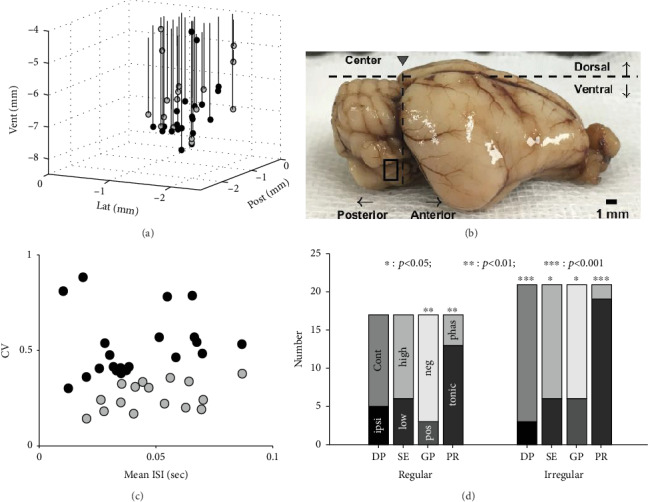
Neural recording locations in three dimensions (3D) in black (irregular) and gray (regular) circles (a). The relative recording positions are determined by the center, which is designated by the cross point of the sagittal suture line and the external auditory canals. The signs for the ventral, the posterior, and the lateral to rightward direction are negatively presented. The rectangle in black indicates the possible region for the vestibular nucleus (VN) (b). The relation between mean interspike interval (ISI) and coefficient of variation (CV) is presented with the normalized coefficient (CV^∗^) in black (>0.15) and gray (<0.15) circles (c). Based on the CV^∗^, the neuronal characteristics, such as directional preference (DP), sensitivity to initial GVS (SE), GVS polarity (GP), and physiological response to GVS (PR), are presented in the stacked histograms with statistical significance (d). Star implies statistical significance in a stacked bar.

Thirty-eight canal-originated neuronal responses were recorded in VN. The presentation in two dimensions (2D) of the examinee's view from the top indicated most positions (35/38, 92.1%) matched with the referenced position, and others (3/38, 7.9%) were also closely (<0.1 mm) located to the reference. On the other hand, the recording locations related with the recording depth had a wider range (4.34-7.89 mm in depth) than that of the reference (6.1-8.3 mm in depth). The locations of the neurons in three dimensions (3D) are presented (Figure 3(a)), and its possible area was marked on the animal's brain (Figure 3(b), rectangle in black). According to the calculated discharge regularity (Equation (2)), 17 neurons were regular in gray circles and 21 were classified as irregular neurons in black circles (Figures 3(a) and 3(c)). The number of neuronal characteristics for each group was summarized in multiple histograms with statistical significances (Figure 3(d)). In the regular neurons, there were five ipsi-DP and six low SE neurons with no statistical significance (*p* > 0.0717). However, most neurons (18/21, 85.7%) in the irregular group were contra-DP neurons with significance (*p* = 7.4482 × 10^−4^). From the aspect of the PR, the tonic neurons were more than phasic in both regular (13/17, 76.5%) and irregular groups (19/21, 90.5%) with significance (*p* < 0.0064) (Figure 3(d)).

**Figure 4:**
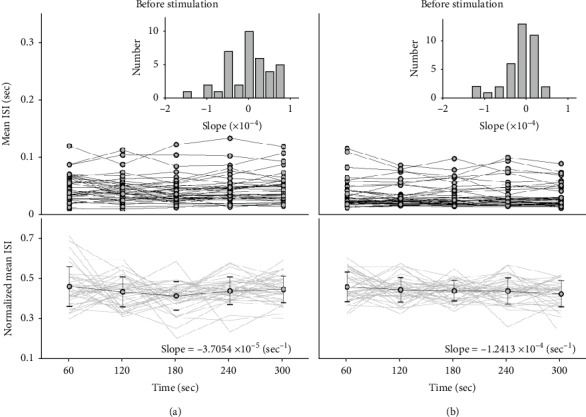
Averaged interspike interval (ISI) before (a) and after (b) stimulations. During one second before and after the repeated GVS (amplitude: -100 *μ*A, time of stimulation: 3 sec) in every 60 sec, the neuronal ISI are presented by their averages. The subplots in the top figures in (a) and (b) indicate the distribution of the slopes based on the averaged ISI. At the bottom figures in (a) and (b), the normalized mean ISI are presented with their average (gray circles) and standard deviation (black lines) for comparing the calculated values. The values in the bottom figures show the computed slopes based on the averaged normalized mean ISI.

For the parameters of GVS, several neurons were selected and tested to avoid the synaptic fatigue (see Galvanic Vestibular Stimulation (GVS)). The longest duration with no synaptic fatigue was 8.7 sec, and the relative short duration (3 sec) of GVS (100 *μ*A) was adopted for no effects on the neuronal responses. In addition, the interval between two successive stimuli was also examined by investigating the recovering time of the averaged IFR after GVS. Based on the selected tests, the averaged IFR was fully recovered in less than 10 seconds after stimulation, and the given interval (60 sec) was enough time for the complete the recovery in IFR before the next stimulation. Thus, the stimulating parameters in this study were 100 *μ*A, 3 seconds, and 60 seconds for the GVS amplitude, the GVS duration, and the interval between two successive GVS, respectively, agreeing with a previous study [35]. For the evaluation of the GVS parameters, the mean ISI of neurons and theirnormalized values were presented before and after stimulation (Figures 4(a) and 4(b)). The information of mean ISI and their normalized mean ISI before stimulation indicated that the repeated GVS rarely changed the mean ISI, resulting in small slopes in both mean ISI (<1.59 × 10^−4^) and averaged normalized mean ISI (−3.71 × 10^−5^ sec^−1^). Similar results were found in the information after stimulation, and the mean ISI and averaged normalized mean ISI were less than 1.42 × 10^−4^ and −1.24 × 10^−4^ sec^−1^, respectively, revealing that the repeated GVS with the applied parameters had little effects on the neuronal ISI.

**Figure 5:**
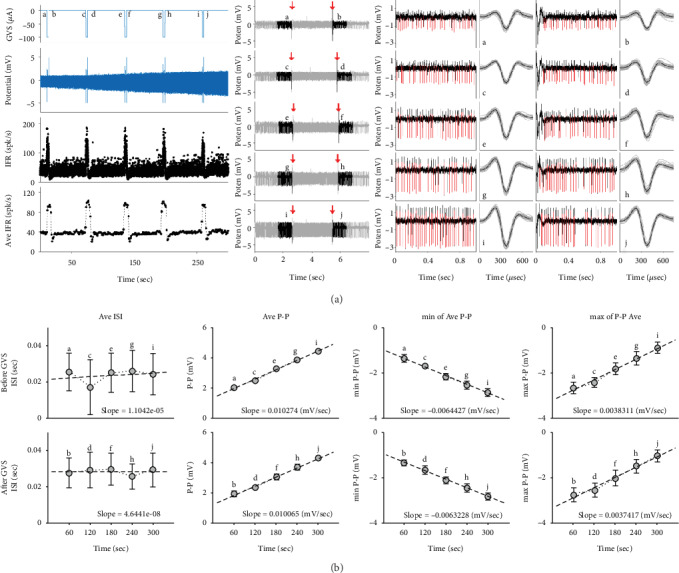
Example of neuronal response to successive GVS. (a) On the left row of the figure, there are the applied stimuli (top) with a constant interval (60 sec), the corresponding neuronal spikes (1^st^ middle) and IFR (2^nd^ middle), and the averaged IFR (bottom). On the second row of the figure, single event (a-b, c-d, e-f, g-h, and i-j) is determined and independently analyzed to identify the change of the potentials during the repeated GVS. Downward arrows indicate the start and the end of GVS. From the initial spikes before and after GVS, the spikes (in red) are sorted to compute the neuronal potentiation (P-P) and interspike intervals (ISI). The third, fourth, fifth, and sixth rows of the figure represent the selected spikes before GVS, the overlapped spikes before GVS, the selected spikes after GVS, and the overlapped spikes after GVS, respectively. The selected spikes are shown in red, and their overlapped spikes are presented in gray with their averages in black. (b) The first row of the figure shows the change of the averaged ISI (gray circle) with its standard deviation (black line) before (top) and after (bottom) GVS. The second, third, and fourth rows of the figure display the change of the averaged potential, its minimum and maximum of the averaged potential, respectively, before (top) and after (bottom) GVS. In all plots, the linear regression (dashed line) is added to explain each change, and their standard deviations (black line) are presented.

A neuronal example responding to five successive stimuli with a constant interval is shown in Figure 5. The applied GVS (top), the neuronal spikes (1^st^ middle), the IFR (2^nd^ middle), and the averaged IFR (bottom) are presented at the first column of Figure 5(a). As a negative GVS was applied, the IFR increased. Also, its DP was contralateral, and the PR of this neuron was tonic. Based on the computation, this example was an irregular (CV^∗^ = 0.31) and a high-sensitive neuron (SE = 1.41). The spikes were examined by the separated events: a-b, c-d, e-f, g-h, and i-j. (see Data Analysis). The averaged P-P increased during both before and after GVS. The P-P before stimulation continuously increased from 2.03 to 4.41 mV (changing rate: 10.3 *μ*V/stimulation) (Figure 5(a), 4^th^ row) and that after the stimulation also increased from 1.95 to 4.30 mV (changing rate: 10.1 *μ*V/trial) (Figure 5(a), 6^th^ row). However, the repeated stimuli rarely affected the neuronal ISI maintaining its averages by 23.5 millisecond before GVS and 28.5 millisecond after GVS (Figure 5(b)). Both increases in the P-P were led by the decreasing minimum and the increasing maximum of the averaged spikes.

**Figure 6:**
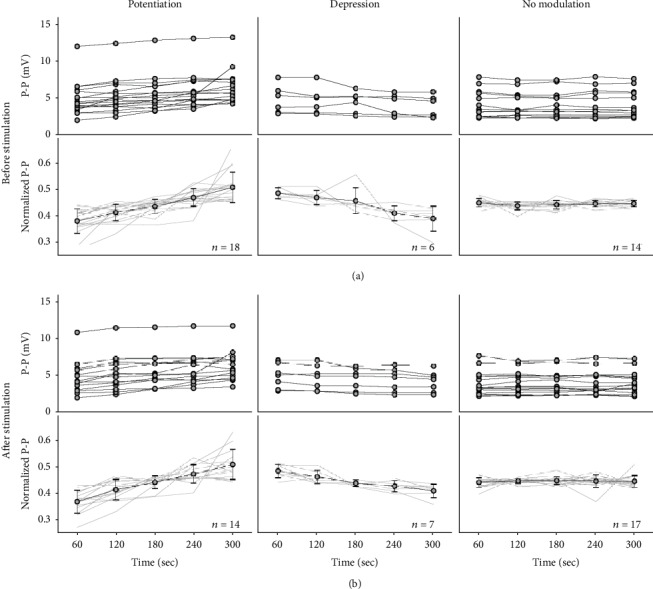
Modification in the potential, the peak-to-peak (P-P), of the spike before (a) and after (b) GVS. Depending on the slope of P-P, the neuronal responses are classified into 3 groups: potentiation, depression, and no modulation. In each figure, the P-P (top) and their normalized values (bottom) are presented, and the average and standard deviation of the normalized values are added. The numbers of the bottom plots in (a) and (b) indicate those of classified neuronal responses for each group before and after stimulation, respectively.

Like the example, almost half of neurons (47.4%, 18/38) increased their P-P as GVS was repeated, and only six neurons (15.8%) decreased the P-P under the GVS repetition before stimulation. Counting them together as the neurons with modulation in the P-P, two-third of neurons (24/38) modulated their P-P before stimulation (Figure 6(a)). After stimulation, the overall number of modulated neurons in the potentials decreased (21/38, 55.3%), showing the 14 increasing (66.7%) and 7 decreasing P-P (33.3%), specifically. According to the number of the modified P-P, the modification before stimulation was significant (*p* = 0.037) while that after stimulation showed no significance (*p* = 0.209) (see Statistical Test). The phenomenon of the neuronal modulation was more emphasized in the normalized P-P. The modulating rates before stimulation were 5.30, -4.29, and -0.04 (×10^−4^/sec^−1^), and those after stimulation were 5.76, -3.15, and 0.13 (×10^−4^/sec^−1^) for the groups of potentiation, depression, and no modulation, respectively (Figure 6(a) and 6(b), 2^nd^ row).

The modulation in P-P was statistically evaluated if any neuronal characteristics, such as DP, GP, SE, and PR, were correlated with the modulation. Of 24 modulated neurons, there were 19 neurons with the contralateral preference (79.2%), showing the statistical significance (*p* = 0.0008) before stimulation. Although the number of the modulated neurons decreased, a similar result for DP was maintained in the same group after stimulation (*p* = 0.0007). In the groups of no modulation before and after stimulation, the neurons with contralateral preference were the major population with significance (*p* = 0.029 and 0.025, respectively). The bias in the neuronal characteristics was statistically identified as summarized in Table 1. In the groups of potentiation before and after stimulation, all characteristics were significantly biased except SE while PR showed a significance (*p* = 0.016) in that of depression before stimulation. The biased population was observed in the group of no modulation before stimulation (*p* < 0.029) except PR. On the other hand, the group of depression after stimulation showed no significance in all characteristics (*p* > 0.063), and all characteristics were biased in that of no modulation after stimulation (*p* < 0.025).

**Figure 7:**
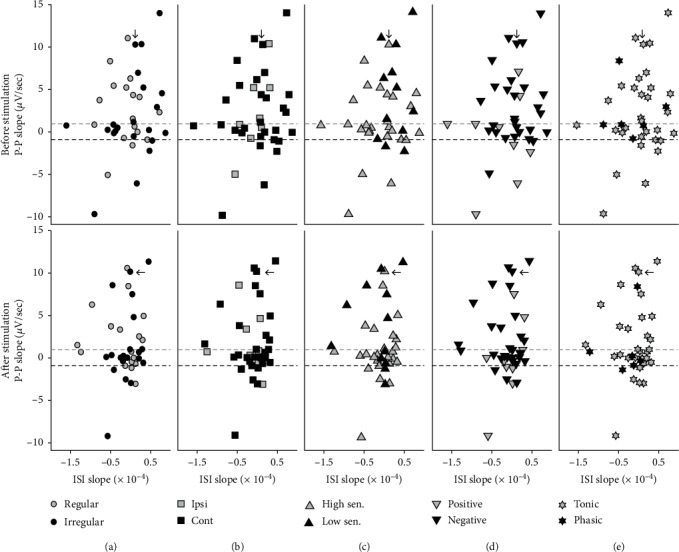
Relations between two responding slopes of peak-to-peak (P-P) and interspike interval (ISI) before (1^st^ row) and after (2^nd^ row) GVS, depending on the neuronal characteristics: the discharge regularity (a), the directional preference (b), the neuronal sensitivity to GVS (c), the polarity of GVS (d), and the physiological response (e). The relations are separated by the neuronal modifications, such as potentiation, depression, and none, in the P-P, and the references for the division are represented by the dashed lines (±1 *μ*V/sec) in all plots. The arrows in the figures designate the characteristics of the example shown in Figure 5.

The relations between two slopes of P-P and ISI under the neuronal characteristics were examined if the responding patterns of the neuronal ISI affected those of P-P before (Figure 7, 1^st^ row) and after (Figure 7, 2^nd^ row) stimulations. In the group of potentiated neurons, the ranges of ISI slopes (range: −0.77 × 10^−4^~0.78 × 10^−4^) before stimulation decreased after stimulation (range: −1.33 × 10^−4^~0.45 × 10^−4^), which reduced based on their averages from 0.12 × 10^−4^ to −0.16 × 10^−4^. A similar phenomenon was shown in the group of depression before and after stimulation (from −0.04 × 10^−4^ to −0.16 × 10^−4^), but that of no modulation increased (from −0.20 × 10^−4^ to −0.03 × 10^−4^). The P-P slopes also changed before and after stimulation, but the increasing or decreasing pattern for each group was not matched to that in the ISI slope. Thus, there were few relations between ISI and P-P based on the comparison of their slopes.

Before stimulation, the parabolic relations were maintained in both regular and irregular neurons while the relations under other neuronal characteristics were not always retained (Figure 7, 1^st^ row). The parabolic relation in the potentiated neurons was shown in the contralateral preferred, the high-sensitive, and the tonic neurons before stimulation. The neurons with the opposite characteristics, such as the ipsilateral preferred, the low-sensitive, and the phasic neurons, had no specific relation between the slopes of ISI and P-P. The linear relation in the no modulated group was shown in the irregular, the contralateral preferred, the high-sensitive, and the tonic neurons. However, the relation in the depressed group was rarely identified due to the lack of data to establish a relation. After stimulation, the parabolic relation was maintained in both regular and irregular groups, but there was no specific relation in the depressed neurons (Figure 7, 2^nd^ row). Also, the linear relation in the no modulated group was barely identified in other characteristic neurons except highly sensitive neurons.

Due to the critical function in plasticity by the glutamate receptors, their numerical change by GVS was examined. The number counts were performed in several selected samples, and it was conducted by separating AMPA and NMDA (see Immunohistochemistry). Both numbers of AMPA (mean ± STD: 36.5 ± 4.45) and NMDA (33.17 ± 4.05) before the stimulation decreased after stimulation, 26.17 ± 3.7 and 27.83 ± 3.02 for AMPA and NMDA, respectively. Based on the counted number of the receptors, the change of AMPA (28.32%) was bigger than that of NMDA (16.09%) (Table 2). However, the current results had a limit to show the direct relation to the modification in the neuronal amplitudes, and the possible limitations were discussed in detail (see Amplitude Modulation and Glutamate Receptors).

## 4. Discussion

This study examined the modulation of the neuronal action potential by the repeated GVS to understand the neuroplasticity in the early stage of the central vestibular system. The modified potential was assessed by probing its relations with the neuronal characteristics. As shown in previous studies, the underlying mechanisms for the neuroplasticity induced by various external stimuli have been examined in different animal models [36–38] while few direct investigations on the modification of the neuronal potential have been conducted. There have been several examinations to elucidate the alterations of the vestibular-related neural information using GVS. According to a previous study, the electrical potentials increased during GVS [21], and the results showed the possibility that the neuronal potentials were modulated by the given stimulation. Other studies examined the improvements in the face perception deficit [39] and the motor functions during GVS [40, 41]. However, all these results showed the functional alterations of the vestibular system instead of examining its neural origin. Even though the neuronal effects by the repeated GVS have been studied with different analyses, these studies focused on the neuronal firing rates during GVS [25, 42].

The modulation of neuronal potential by the electrical stimulation has been hardly observed because of the high voltage of the stimulus [43, 44]. To overcome this problem, the neuronal potentials were collected in every 1 second before and after the repeated stimuli in this study (see Data Analysis). In addition, the modulation of the neuronal potentials was analyzed by normalizing the recorded data to emphasize the modulation. This approach clarified the change in the neuronal potentials as well as visually enhanced the responding pattern by the repeated stimulations (Figure 6). Even though no concurrent neuronal activities during the stimulation were measured, the remaining effects by the stimulation in the neuronal potentials could be identified based on the method used in this study.

### 4.1. Potentiation and Depression Had No Relation with ISI

The instantaneous firing rate (IFR) was calculated by the reciprocal of the ISI between two successive neuronal spikes, and vice versa. Generally, the firing rate (FR) of a neuron encodes the neuronal response to an incoming stimulus. Even though the neuronal responses could vary even under the same stimulus [26], the concurrent FR was a sustainable indicator to represent the effects by the stimulation. Unlike the FR, on the other hand, the amplitude of potential (P-P) was similarly maintained during the neuronal recording while the distance between the measuring electrode and the targeted neuron was preserved. However, the P-P tended to be reduced and the FR increased during GVS during the stimulation, which possibly suggested that the potential modulation might be affected by the change of the ISI. To elucidate this possibility, the ISI before and after GVS was examined for the direct correlation between the ISI and its relevant potentials. The effects on the potential modulation by the change of the ISI were inspected through the relations between the ISI and the P-P, and our current study examined them by applying a linear regression (see Statistical Test). Based on the analysis, only a few neurons (2/38, 5.3%) showed a significant linear relation between the ISI and the P-P both before and after stimulations (R-squared: >0.95 and >0.86, respectively), indicating that the change in the ISI rarely affected the potential modulations. Similar results were maintained in the separated relations before and after stimulations (R-squared: >0.71 and >0.74, respectively), showing only four neurons (10.5%) both before and after GVS had the linear relations. The current results demonstrated the linearity between the ISI and the P-P was weak with no significance in the population, and the results suggested that the potential modulation was rarely affected by the that of the ISI.

### 4.2. The Change in the Number of Glutamate Receptors by the Repeated Electrical Stimulation

The glutamate receptors have known to be the important channels for the neural communication, and AMPA and NMDA have been considered as the core neurotransmitters for synaptic plasticity [35, 45]. The current results showed that the changes in the number of glutamate receptors were induced by the repeated electrical stimulation in VN, agreeing with previous studies [23, 46, 47]. The dominant modification of synaptic strength, such as potentiation or depression, was mainly regulated by the postsynaptic glutamate receptors [46]. However, it was still elusive what kind of receptors mainly led the dominant changes in the synaptic strength that demonstrated the plasticity. According to the current results in this study, it was clear that the repeated electrical stimulation affected the neuronal potentials, and the changed number in the glutamate receptors. Unfortunately, however, their relations were unclear to show that the changes in the neuronal potential and the number of the receptors were closely related before or after stimulation. Simply, the electrical stimulation modified the neuronal potential as well as the number of glutamate receptors, and the concurrent outcomes under the same stimuli might have a close relation with the synaptic plasticity. The plasticity by the glutamates is still debated. Even though some studies insisted that the number of AMPA changed more than that of NMDA in plasticity [34, 48, 49]. For the better insights on this underlying mechanism, more supplementary results are required by providing direct experimental outcomes. Therefore, our current results had a limit to explain the relation between the neuronal potential and the number of glutamate receptors by the stimulation.

### 4.3. Amplitude Modulation and Glutamate Receptors

Vestibular functions are achieved through the summation of the neuronal responses to an incoming stimulus, and the responses are generated by the diverse neurotransmitters [50]. Of these neurotransmitters, the glutamate is one of the most common types, and its relevant receptors provide a strong uptake mechanism. Although the effect is sometimes revealed as an inhibition [51], the glutamate is generally categorized as an excitatory neurotransmitter. Based on the accumulated evidences, the neural effects by glutamates are mainly controlled by the activation of the related receptors [52]. In various subtypes of glutamate receptors, AMPA and NMDA receptors are widely known to play a critical role for the neural plasticity [46, 48, 53], and their colocalization in VN has been identified in previous study [54]. The depolarization of a neuronal potential is initiated by the agonist binding of AMPA, opening the channels for the ions of sodium (Na^+^), potassium (K^+^), and calcium (Ca^2+^). While the initial is dominantly led by AMPA, the prolonged depolarization continues by NMDA [55]. Once the potential reaches at a certain level (~30 mV), the open channels close to prevent the additional flow of the positive ions into the intracellular area. However, the dominance between AMPA and NMDA on the synaptic plasticity is still under debate, weighting on NMDA [34, 46, 48, 55]. In the current results, the neuronal potential was modulated as increased or decreased by the repeated GVS (Figure 6). As described, the combined effect by AMPA and NMDA produces the modulation in the potential amplitudes, and the change of their relevant receptors is one of the direct contributors. Thus, two consequences, such as the modulation in the amplitude and the change in the number of the glutamate receptors, suggested their physiological link by the repeated GVS.

However, the attainable relation is still limited by two aspects. First, the selected histological images to count the number of receptors were possibly biased. To avoid this problem, the images were randomly selected, and the receptor counting for each image was performed independently (see Immunohistochemistry). Nevertheless, there remains the issue of the biased selection, and it might influence the obtained quantitative information. For the better quantitative assessment in 2-dimensional (2D) morphometry, previous studies suggested a stereological approach [56, 57]. In the stereological method, the bias in the assessment was avoided mainly by the systemic uniform random sampling (SURS). Unlike a simple random sampling as done in this current study, SURS uses multiple 2D structures apart by the same distance [58]. Through this method, SURS avoids the sampling bias. Second, the same neuron, which modified its amplitude in vivo, was not possible to be identified on the sectional area in vitro. This was the similar issue in the previous study [23]. However, the study demonstrated the relation between the repeated electrical stimulation and the increase in the number of glutamate receptors, concluding the results as a long-term potentiation (LTP) [23]. In summary, a potential relation between the modulation in the neuronal amplitude and the change in the number of the glutamate receptors would be clarified by resolving these limitations.

## 5. Conclusion

The neuronal potential modulation in VN was examined as GVS was repeatedly applied. The analysis of the neuronal amplitudes indicated that a significant number of neurons changed their amplitudes by the repeated GVS, and the statistical test on the potentials demonstrated the modulation was generated by the applied stimulation. Considering the direct effects on the neuronal potentials by the electrical stimulation, the potential modification was related with the synaptic strength, suggesting the synaptic plasticity in VN. Therefore, the neural modification for the plasticity was generated in the early central stage of the vestibular system by the repeated GVS, and it was assessed by the potential modification in multiple neuronal spikes.

## Figures and Tables

**Table 1:** Statistical evaluation of P-P modulation based on neuronal characteristics.

	Before stimulation						After stimulation					
	Pot		Dep		No mod		Pot		Dep		No mod	
Total	18		6		14		14		7		17	
DP	ipsi	con	ipsi	con	ipsi	con	ipsi	con	ipsi	con	ipsi	con
	4	14	1	5	3	11	3	11	1	6	4	13
*p*	0.0154		0.1094		0.0287		0.0287		0.0625		0.0245	

GP	pos	neg	pos	neg	pos	neg	pos	neg	pos	neg	pos	neg
	2	16	4	2	3	11	2	12	4	3	3	14
*p*	0.00065613		0.3438		0.0287		0.0065		0.5		0.0064	

SE	Low	High	Low	High	Low	High	Low	High	Low	High	Low	High
	8	10	2	4	2	12	7	7	2	5	3	14
*p*	0.4073		0.3438		0.0065		0.6047		0.2266		0.0064	

PR	ton	phas	ton	phas	ton	phas	ton	phas	ton	phas	ton	phas
	16	2	6	0	10	4	13	1	5	2	14	3
*p*	0.00065613		0.0156		0.0898		0.00091553		0.2266		0.0064	

Pot=potentiation; Dep=depression; No mod=no modulation; DP=directional preference; GP= GVS polarity; SE=Sensitivity; PR=physiological response; *p*=*p*-value; ipsi=ipsi-preferred; con=contrapreferred; pos=positive; neg=negative; low=low-sensitive; high=high-sensitive; ton=tonic; phas=phasic.

**Table 2:** Summary of the counted receptors of AMPA and NMDA.

Receptor	AMPA				NMDA			
Stimulation (condition)	Before (control)		After (stimulated)		Before (control)		After (stimulated)	
Side	Left	Right	Left	Right	Left	Right	Left	Right
Area #1	43	40	21	24	36	39	30	27
Area #2	37	32	23	31	29	33	26	32
Area #3	38	29	28	30	32	30	23	29
Average (±STD)	39.33 (±3.21)	33.67 (±5.69)	24 (±3.61)	28.33 (±3.79)	32.33 (±3.51)	34 (±4.58)	26.33 (±3.51)	29.33 (±2.52)
Total	36.5 ± 4.45		26.17 ± 3.7		33.17 ± 4.05		27.83 ± 3.02	
Change in %	28.32				16.09			

## Data Availability

The data in this study are included within the article.
